# Dynamic Onset of Feynman Relation in the Phonon Regime

**DOI:** 10.1038/srep25690

**Published:** 2016-05-09

**Authors:** Y. Li, C. J. Zhu, E. W. Hagley, L. Deng

**Affiliations:** 1 Department of Physics, East China Normal University, Shanghai 200241, China; 2 School of Physical and Engineering Sciences, Tongji University, Shanghai 200092, China; 3 National Institute of Standards and Technology, Gaithersburg, Maryland 20899, USA

## Abstract

The Feynman relation, a much celebrated condensed matter physics gemstone for more than 70 years, predicts that the density excitation spectrum and structure factor of a condensed Bosonic system in the phonon regime drops linear and continuously to zero. Until now, this widely accepted monotonic excitation energy drop as the function of reduced quasi-momentum has never been challenged in a spin-preserving process. We show rigorously that in a light-matter wave-mixing process in a Bosonic quantum gas, an optical-dipole potential arising from the internally-generated field can profoundly alter the Feynman relation and result in a new dynamic relation that exhibits an astonishing non-Feynman-like onset and cut-off in the excitation spectrum of the ground state energy of spin-preserving processes. This is the first time that a nonlinear optical process is shown to actively and significantly alter the density excitation response of a quantum gas. Indeed, this dynamic relation with a non-Feynman onset and cut-off has no correspondence in either nonlinear optics of a normal gas or a phonon-based condensed matter Bogoliubov theory.

The Feynman relation^1^, originally derived to describe the density excitation spectrum of superfluid ^4^He at *T* = 0, provides a very fundamental understanding of the collective response of an ultra-cold gaseous or solid-state system in the small phonon regime without spin changes. The most celebrated predictions of this important relation are the linear dependency of quasi-momentum transfer in the excitation spectrum of the ground state when the external interaction is neglected, and the corresponding behavior of the system structure factor. Indeed, such monotonic behavior approaching zero excitation energy as the quasi-momentum transfer reduces has been widely accepted in solid-state physics^2^.

Nonlinear optics^3^, a completely non-related field of study, investigates a wide range of light-matter interactions from sub-atomic particles and condensed matter physics^4^,^5^,^6^, to astrophysical phenomena^3^,^7^. Although widely used in many fields of physical science, nonlinear optics usually only serves as an indispensable probe (especially when the light intensity is not very high) rather than a tool to actively and dynamically alter the fundamental properties of a material under investigation except the intensity-dependent effects induced by ultra-high power ultra-short-pulse lasers^3^,^8^. The discovery of gaseous phase Bose-Einstein condensates^1^, now referred to as bosonic quantum gases^9^, has significantly changed our understanding of nonlinear optics of light-matter interactions, even at very weak field strengths. Surprisingly, the non-linear optical response of quantum gases can be fundamentally different from that of normal gases^10^. Indeed, many effects and phenomena well-known to nonlinear optics in normal gases are now subject to significant modification and often require a completely different interpretation. Moreover, many nonlinear wave-mixing processes in normal gases with well-understood physics are now found to have no correspondence in quantum gases.

Here we show how nonlinear optics of a weak Sum-Frequency-Generation (SFG) process can profoundly impact the collective response of a bosonic quantum gas in a spin-preserving process. We show that even a weak light-matter wave-mixing process in a quantum gas can significantly alter the well-known single-spin Feynman relation in the phonon regime, resulting in a dynamic non-Feynman onset and cut-off in the ground state excitation spectrum. This is a profoundly fundamental change because (1) never in the history of condensed matter physics has the single-spin Feynman relation in the phonon regime been challenged; and (2) never before has a nonlinear optical process been shown to have such a profound impact on both the condensed-matter collective response of the system and the physics of the light-field generation process. These dynamic effects may open many possibilities for novel nonlinear optical processes in quantum gases.

## Results

### Model

**Figure 1:**
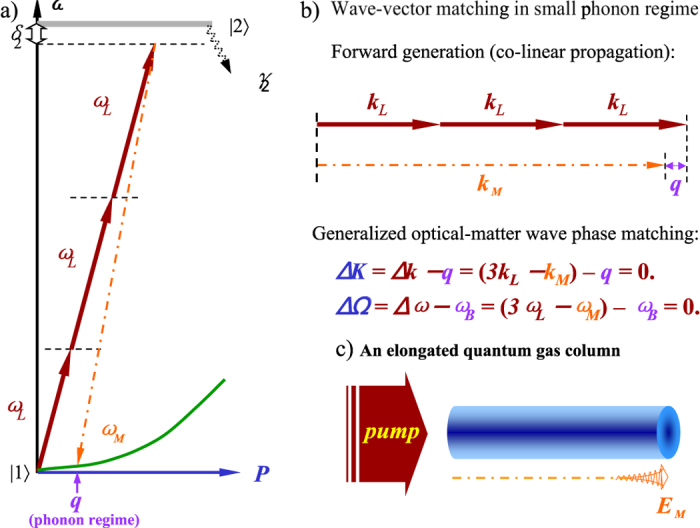
Optical-matter wave-mixing in a quantum gas. (**a**) Energy level diagram and laser couplings for a SFG process in a quantum gas. The dash-dotted arrow denotes the forward emission at *ω*_*M*_ in the Feynman small phonon regime (small *q*). Resonant and off-resonant backward emission processes only occur in the free-particle regime (large *q*) and have been neglected. (**b**) Wave-vector diagrams and the generalized optical-matter wave phase-matching conditions. (**c**) Excitation geometry.

We begin by considering an elongated Bose condensate with its long axis aligned with the *z*-axis (Fig. 1).

We excite the condensate with a pump laser field E_*L*_ with wave vector k_*L*_ that is linearly polarized along the *x*-axis and propagates along the *z*-axis. Because of the allowed dipole coupling between states 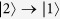 an SFG field E_*M*_ with wave vector k_*M*_ and frequency *ω*_*M*_ is generated from electronic state 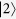 (Fig. 1a).

The Gross-Pitaevskii equation^11^,^12^ describing the evolution of the atomic mean-field wave function in the presence of the pump field is given by


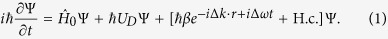


Here, 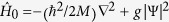 is the trap-free^13^,^14^ system Hamiltonian without the external light field and 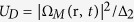 describes the optical-dipole potential arising from the internally-generated SFG field. 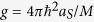 with *a*_*S*_ and *M* being the s-wave scattering length and the atomic mass, respectively. 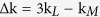 and 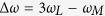 are the usual optical-wave vector and energy mismatch. Together they describe the phase-mismatch between the pump and the internally-generated fields. We have defined 
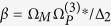
, where 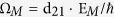 is the Rabi frequency of the generated field and 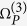 is the effective three-photon Rabi frequency of the pump field. d_21_ is the dipole transition operator between states 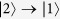 and 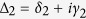, where 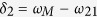 is the detuning of the generated field from the electronic state 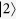 having a resonant line width of *γ*_2_.

The critical element that distinguishes the Hamiltonian in Eq. (1) from the Hamiltonian describing light-matter multi-wave mixing in a quantum gas^10^ is the dipole potential energy term 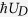 on the right side of Eq. (1). This term arises from the internally-generated SFG field but has been neglected in all light-quantum gas studies reported to date. However, we show here that this term is a vitally important element in nonlinear optical properties of light-matter interactions in the phonon regime; the regime where the well-known Feynman relation dominates. It is important to emphasize that *U*_*D*_(r, *t*) is a *dynamically* changing quantity depending on the generation and coherent propagation of the wave-mixing field Ω_*M*_(r*, t*), and hence it cannot be considered as a *static* trap potential in 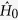. In fact, it can be shown mathematically that any quasi-static external trapping mechanisms, magnetic or optical, can be removed from the Maxwell equation for the SFG field by a phase transformation and therefore have no effect on the Feynman relation in the phonon regime.

In the single-spin Feynman phonon response regime no trap potential exists^13^,^14^, and the excitation of the system is described by small quasi-momentum transfer q usually arising from thermal agitations. This trap-free Hamiltonian corresponds to an atomic Bose-Einstein condensate system where the trapping potential is fully turned off. This avoids the initial mean-field reaction that completely masks the small phonon regime in which the Feynman relation applies. This is exactly what has been done experimentally in measurements of the structure factor of a Bose-condensate^13^,^14^. It is then immediately clear that with the external potential an *additional* small, dynamic and yet *negative* excitation energy can profoundly alter the energy spectrum and response of the system. This is achieved by an optical wave-mixing process with a *negative detuning* which results in a dynamic internally-generated field and a *non-adiabatic* dipole potential *U*_*D*_(r*, t*) < 0 that can cancel the phonon energy in the excitation spectrum and thereby drastically change the Feynman relation.

### Maxwell-Bogoliubov theoretical framework

With the above general argument we begin our calculation using the Maxwell-Bogoliubov theoretical framework for quantum gases^10^. We generalize the seminal study of Raman wave mixing and scattering by Bloembergen and Shen^15^,^16^ to encompass both atomic center-of-mass (CM) motion and density excitations required for a quantum gas.

We assume that the Bose-Einstein condensate wave function of a single-specie is given by


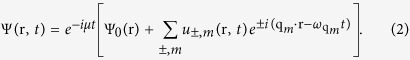


Here, Ψ_0_(r) is the ground state condensate wave function in the absence of any external light fields and 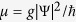 is the chemical potential. In addition, 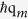 and 
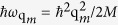
 are the quasi-momentum transfer and the energy of the elementary excitation induced by the light-wave mixing and scattering process, respectively, with *m* being the Bogoliubov excitation mode index. For mathematical simplicity and without loss of generality, we only consider the lowest Bogoliubov mode by neglecting the mode index *m*. Multi-Bogoliubov modes can be similarly solved analytically. The effect is just a slight broadening of the width of the SFG field.

Defining 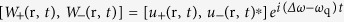 and substituting Eq. (2) into Eq. (1) we obtain^1^


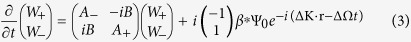


where 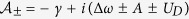, 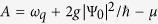, 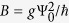, and 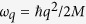. The generalized optical-matter wave vector and energy mismatch 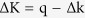 and 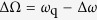 encompass both optical-wave and fundamental excitations. We have also introduced a phenomenological motional state resonance line width *γ* which characterizes the damping of the elementary excitation^17^.

Under the slowly varying envelope approximation the Maxwell equation for the wave-mixing field E_*M*_ propagating along the *z*-axis (forward direction) can be written as^10^,^18^


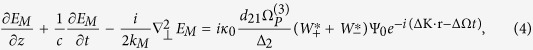


where 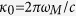. Mathematically, Eq. (3) can be formally integrated and inserted into the right side of Eq. (4) from which the propagation properties of the wave-mixing field can be numerically evaluated. For mathematical simplicity, and for the purpose of demonstrating the key underlying physics, we seek without the loss of generality a first-order solution of Eq. (3) that is adiabatic with respect to optical response but non-adiabatic with respect to atomic center-of-mass motion. The non-adiabaticity with respect to the atomic CM motion reflects the fact that *U*_*D*_ cannot be treated as a static trap potential, as discussed before. We emphasize, however, that we have solved Eqs. (3, 4) numerically without any approximation and obtained the same results. With the above approximations we obtain from Eq. (3)


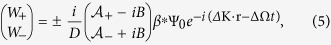


where 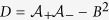. Using Eq. (5) to construct the polarization source term for the SFG field, we obtain the Maxwell-Bogoliubov equation


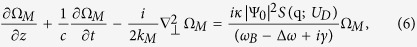


where the Bogoliubov fundamental excitation spectrum *ω*_*B*_(q; *U*_*D*_) and the quantum gas structure factor *S*(q; *U*_*D*_) are given by


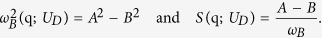


In deriving the above results we have defined 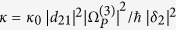, enforced the total optical-matter wave phase matching in the forward direction, and also neglected far-off resonance contributions.

Under the lowest-order approximation in the phonon regime, the above expressions for the excitation spectrum and the structure factor of the quantum gas become


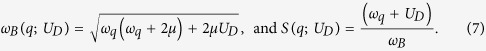


Clearly, when *U*_*D*_ is neglected Eq. (7) reduces to the well-known Feynman variational approximation^1^ for the density excitation spectrum of superfluid ^4^He at *T* = 0. We emphasize that Eqs. (6, 7) are obtained within standard nonlinear optics formalism 3, and are therefore completely unrelated in anyway to the local density approximation treatment.

### Bogoliubov excitation spectrum and condensate structure factor

**Figure 2:**
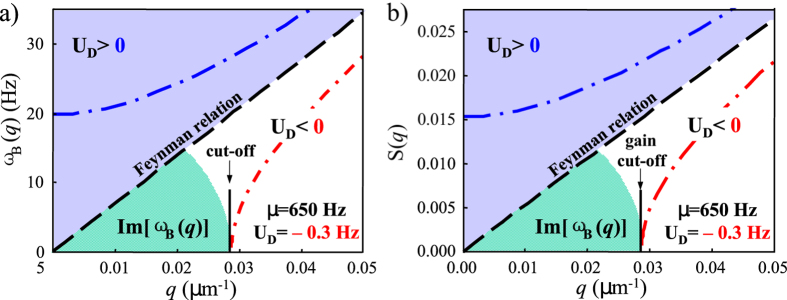
Fundamental excitation energy and quantum gas structure factor in the phonon regime. Bogoliubov excitation spectrum *ω*_*B*_(*q*) [plot (**a**)] and the condensate structure factor *S*(*q*) [plot (**b**)] exhibit a dynamic non-Feynman onset and cut-off as functions of *q* and *U*_*D*_. Black dashed-lines: prediction of the well-known Feynman relation in the small phonon regime. Red dot-dashed curve: red-detuned pump exhibits a dynamic non-Feynman onset and cut-off. The gray-shaded regions above the Feynman relation are accessible with a Bosonic quantum gas only when the pump is blue-detuned and the photo-de-association time is longer than the bare atomic spontaneous emission time^22^,^23^,^24^. The green-shaded areas indicate regions in which the Bogoliubov spectrum with a red-detuned pump becomes imaginary (forbidden region).

Equation (7) predicts a novel and surprising feature never before seen in nonlinear optics. With a red-detuned pump *δ*_2_ < 0 and *U*_*D*_ = |Ω_*M*_|^2^/*δ*_2_ < 0, the Bogoliubov excitation spectrum for elementary excitations in the wave-mixing process is *dynamically* red-shifted, resulting in a dynamic onset and cut-off in the well-known “static” Feynman relation (Fig. 2a). Correspondingly, the frequency of the generated field *ω*_*M*_ will be dynamically blue-shifted from it original frequency (since 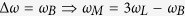). Accompanying this dynamic change in the Feynman relation is an abrupt drop in the quantum gas structure factor in the small quasi-momentum transfer regime (Fig. 2b), resulting in strong suppression of the forward light-wave-mixing and coherent propagation growth process. Indeed, this forward suppression is much more severe and abrupt than predicted by the usual “static” Feynman relation of single spin. The range of *q* < *q*_*c*_ (here 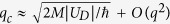 is the critical *q* at which the Bogoliubov dispersion becomes imaginary) forms the region in which wave propagation is forbidden. We note that such cut-offs in the excitation spectrum have been predicted for a Spin-Orbit Coupled (SOC) spinor Bose condensate^19^,^20^ where spin-flip interactions introduce unstable branches which result in such a forbidden regime. In our case, however, the multi-optical wave-mixing process preserves the single-spin state since the cut-off is introduced by nonlinear optical process that are spin preserving. The dynamic feature associated with the wave generation and propagation in such a spin-preserving process has no correspondence with the usual SOC processes which are, in general, instantaneous.

**Figure 3:**
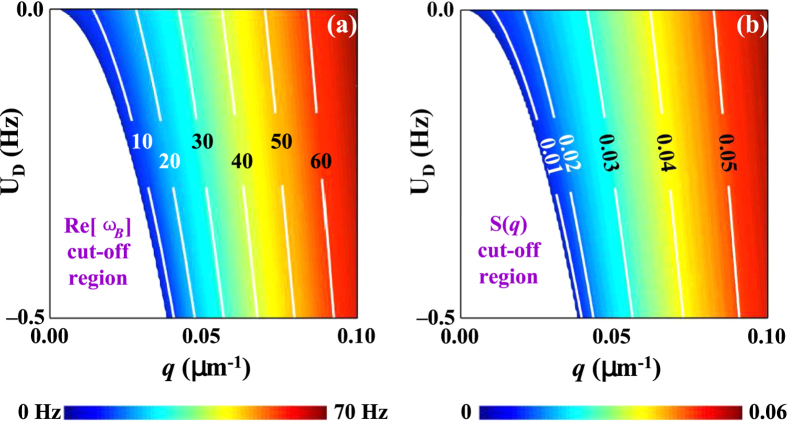
Forbidden regimes of the Bogoliubov excitation spectrum *ω*_*B*_(*q*; *U*_*D*_) and condensate structure factor *S*(*q*; *U*_*D*_). Contour plots of the Bogoliubov excitation spectrum *ω*_*B*_(*q*) (**a**) and the condensate structure factor *S*(*q*) (**b**) as functions of *q* and *U*_*D*_ in the phonon regime. The white areas are regions forbidden by the dynamic non-Feynman cut-off.

Figure 3 displays contour plots of the Bogoliubov excitation spectrum *ω*_*B*_(*q*; *U*_*D*_) and the quantum gas structure factor *S*(*q*; *U*_*D*_) as functions of the optical-dipole potential induced by the generated field and the quasi momentum transfer in phonon regime using Eq. (7). In this small phonon regime where the static Feynman relation dominates the generated field propagates co-linearly with the pump laser. The modified Feynman relation results in a much stronger suppression of the forward wave-mixing gain.

### Dynamical evolution of the forward-generated field

**Figure 4:**
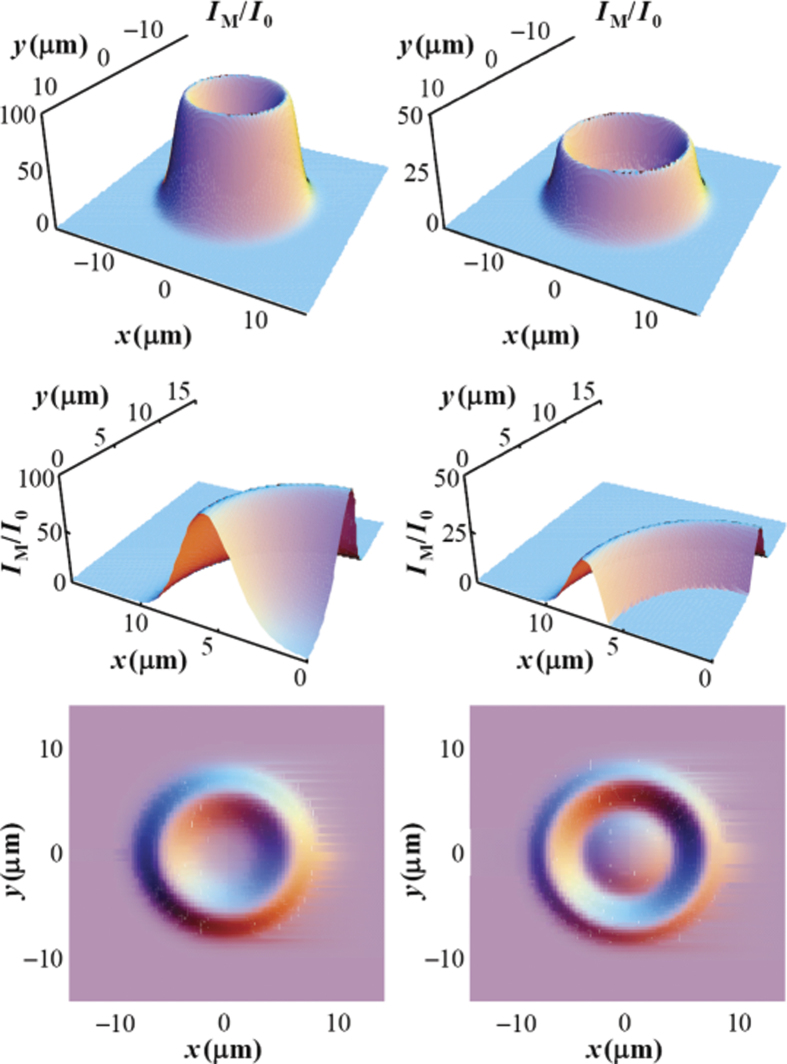
Intensity distribution of the forward-generated field in the phonon regime. Here, we integrate Eq. (6) using Eq. (7). Left column (side-view, center-cut-view, and top-view): the effect of *U*_*D*_ is neglected from Eq. (7). The middle plot clearly shows the linear behavior near the center, as expected from the well-known Feynman relation in the phonon regime. Right column (side-view, center-cut-view, and top-view): the effect of *U*_*D*_ is included in Eq. (7). The presence of a non-Feynman onset and cut-off with a red-detuned pump and SFG field propagating in the forward direction can be clearly seen in the middle plot.

The consequences of the Bogoliubov frequency red-shift can be further investigated and verified numerically by integrating the Maxwell-Bogoliubov equation (6) for the SFG field under the condition of total optical-matter wave phase-matching, i.e., 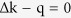 and 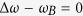. In Fig. 4 we show the transverse distribution of the intensity |Ω_*M*_|^2^ obtained by direct numerical integration of Eq. (6) using Eq. (7). The initial condensate wave function is assumed to have a transverse Thomas-Fermi distribution, i.e., 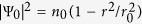 where *n*_0_ ≈ 10^11^/ cm^3^ is the peak condensate density and *r*_0_ is the transverse Thomas-Fermi radius. The initial condition for the generated field is assumed to be 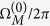 = 3 kHz (corresponding to one initial photon with a pulse duration of 200 *μ*s traveling along the long axis of the condensate having a diameter of 10 *μm*). We take *δ*_2_/2*π* = −1 GHz, *μ *= 600 Hz, *k*_*L*_ = 8.06 *μ*m^−1^, 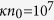 (cm · s)^−1^, *γ*/2*π *= 10 kHz, and *L* = 0.02 cm. The presence of the dynamic non-Feynman onset and cut-off can be clearly seen (plots in the right column) when compared with the results where the small, non-adiabatic, and dynamic effects arising from the internally-generated field are neglected (plots in the left column). Note that in this forward wave generation direction, which is the most efficient wave-mixing and propagation direction in a normal gas, the generated field is suppressed much more strongly than the linear behavior predicted by the well-known Feynman relation. The dynamic non-Feynman onset and cut-off lead to a unique suppression in the structure factor and the coherent propagation gain of the quantum gas that has no correspondence in the nonlinear optical response of normal gases and solid-state materials.

## Discussion and Conclusion

Nonlinear optics of quantum gases is a fascinating new research field in which many new unexpected effects occur that might otherwise be strictly forbidden in normal gases or solid-state materials. Fundamental changes to the single-spin Feynman relation and the nonlinear optical response shown in this work exemplify the novelty of this new research direction within the discipline of nonlinear optics^21^. The exotic new effects and features shown in this study significantly enrich our fundamental understanding of the nonlinear optical response of these intriguing materials referred to as quantum gases. Indeed, none of these novel effects can be obtained by the so-called “matter-wave grating” or “matter-wave superradiance” theory which is fundamentally incapable of explaining any requisite details of light-matter wave-mixing processes in quantum gases^21^.

## Additional Information

**How to cite this article**: Li, Y. *et al*. Dynamic Onset of Feynman Relation in the Phonon Regime. *Sci. Rep*. **6**, 25690; doi: 10.1038/srep25690 (2016).
